# Primary recovery of hyaluronic acid produced in *Streptococcus equi* subsp. *zooepidemicus* using PEG–citrate aqueous two-phase systems

**DOI:** 10.1186/s13568-021-01287-5

**Published:** 2021-08-30

**Authors:** Miguel Flores-Gatica, Héctor Castañeda-Aponte, Mónica Rebeca Gil-Garzon, Liliana Monserrath Mora-Galvez, Martin Paul Banda-Magaña, Jesús Antonio Jáuregui-Jáuregui, Mario A. Torres-Acosta, Karla Mayolo-Deloisa, Cuauhtemoc Licona-Cassani

**Affiliations:** 1 Tecnologico de Monterrey, Escuela de Ingeniería y Ciencias, Centro de Biotecnología-FEMSA, Av. Eugenio Garza Sada 2501 Sur, 64849 Monterrey, NL Mexico; 2 Tecnologico de Monterrey, Núcleo de Innovación de Sistemas Biológicos, Centro de Biotecnología-FEMSA, Av. Eugenio Garza Sada 2501 Sur, 64849 Monterrey, NL Mexico; 3 BIOMENTUM SAPI de CV, Santa María 3050, Vallarta Norte, 44690 Guadalajara, Jalisco Mexico; 4 Tecnologico de Monterrey, Departamento de Bioingeniería, Av. General Ramón Corona 2514, C.P. 45201 Zapopan, JAL Mexico; 5 Department of Biochemical Engineering, The Advanced Centre for Biochemical Engineering, University College London, Torrington Place, London, WC1E 6BT UK

**Keywords:** Hyaluronic acid, Primary recovery, Aqueous two-phase systems, Downstream process, *Streptococcus equi* subsp. *zooepidemicus*

## Abstract

Given its biocompatibility, rheological, and physiological properties, hyaluronic acid (HA) has become a biomaterial of increasing interest with multiple applications in medicine and cosmetics. In recent decades, microbial fermentations have become an important source for the industrial production of HA. However, due to its final applications, microbial HA must undergo critical and long purification processes to ensure clinical and cosmetic grade purity. Aqueous two-phase systems (ATPS) have proven to be an efficient technique for the primary recovery of high-value biomolecules. Nevertheless, their implementation in HA downstream processing has been practically unexplored. In this work, polyethylene glycol (PEG)–citrate ATPS were used for the first time for the primary recovery of HA produced with an engineered strain of *Streptococcus equi* subsp. *zooepidemicus*. The effects of PEG molecular weight (MW), tie-line length (TLL), volume ratio (V_R_), and sample load on HA recovery and purity were studied with a clarified fermentation broth as feed material. HA was recovered in the salt-rich bottom phase, and its recovery increased when a PEG MW of 8000 g mol^−1^ was used. Lower V_R_ values (0.38) favoured HA recovery, whereas purity was enhanced by a high V_R_ (3.50). Meanwhile, sample load had a negative impact on both recovery and purity. The ATPS with the best performance was PEG 8000 g mol^−1^, TLL 43% (w/w), and V_R_ 3.50, showing 79.4% HA recovery and 74.5% purity. This study demonstrated for the first time the potential of PEG–citrate ATPS as an effective primary recovery strategy for the downstream process of microbial HA.

## Key points

- In PEG–citrate ATPS, HA is recovered in the salt-rich bottom phase.
- High PEG MW and low V_R_ are key parameters that promote HA recovery.
- V_R_ 3.50 increases HA purity recovered from a cell-free fermentation broth.

## Introduction

The global hyaluronic acid (HA) market size was valued at USD 9.1 billion in 2019, and it is expected to rise mainly due to increasing aesthetic consciousness and aging populations (Grand View Research 2020). HA is a natural, high molecular weight (10^5^ to 10^7^ Da) linear polysaccharide (Toole 2002). It belongs to the class of glycosaminoglycans and is formed by repeating units of d-glucuronic acid and d-*N*-acetylglucosamine, linked by alternate β-1,4 and β-1,3 glycosidic bonds (Stick and Williams 2009). HA is a main component of the extracellular matrix (ECM), making it a ubiquitous substance. However, it is found in high concentrations in connective tissue, such as hyaline cartilage and skin dermis, and specialised body fluids, like the vitreous humour of the eye and synovial fluid (Falcone et al. 2006). HA plays several roles in the body, such as providing a supportive structure for the cells, controlling tissue hydration and repair, viscoelasticity, and cellular signalling (Cowman and Matsuoka 2005).

HA has become a fascinating biomaterial with diverse applications in medicine, cosmetics, and food, owing to its viscoelastic properties, water retention capacity, biocompatibility, biodegradability, and non-immunogenicity (Sudha et al. 2014). The uses of HA-based products in the medical and cosmetical field include viscosupplementation for arthritis, ophthalmic surgery, prevention of post-surgical adhesion, drug-delivery systems, scaffolds for tissue engineering, wound healing, dermal fillers, and skin moisturisers (Bukhari et al. 2018; Huang and Huang 2018; Mero and Campisi 2014; Price et al. 2007). The final application of HA is dependent on the molecular weight of the polymer, with high molecular weight HA (> 2 MDa) preferred in medicine, whereas a low molecular weight HA (0.8–800 kDa) is preferred in cosmetics (Ghodke et al. 2018).

Large-scale production of HA is accomplished by extraction from animal sources, mainly rooster combs, bovine cartilage, synovial fluids, and vitreous humour (Vázquez et al. 2010). However, HA isolated from these sources are prone to contamination by proteoglycans derived from the ECM, which may represent potential allergens if they are not removed from the final HA product (Murado et al. 2012). Due to immunogenicity concerns, inconsistency in product quality, and the costs of animal-derived HA, microbial fermentation has become a feasible option for industrial production of HA in the last two decades (Sze et al. 2016). Moreover, microbial fermentation can produce HA with specific characteristics such as a specific molecular weight (MW). Microorganisms that naturally synthesise HA, such as *Streptococcus equi* subsp. *zooepidemicus*, or heterologous expression systems, such as *Bacillus subtilis*, *Pichia pastoris*, *Lactococcus lactis*, and *Corynebacterium glutamicum* have been genetically or metabolically engineered to develop strains that produce high molecular weight HA and increased product yield (Chen et al. 2009; Cheng et al. 2019; Jeong et al. 2014; Jia et al. 2013; Kaur and Jayaraman 2016; Wang et al. 2020; Widner et al. 2005).

HA, either animal- or microbial-derived, must undergo meticulous purification processes to obtain a highly pure product that meets the specifications for clinical and cosmetic applications. For microbial HA, the downstream process often involves a combination of alcohol precipitation, adsorption on silica gel, and/or activated charcoal and diafiltration steps (Patil et al. 2011; Rangaswamy and Jain 2008). Proteins are the main impurities present in microbial HA (Cavalcanti et al. 2020), but endotoxins from pathogenic bacteria, such as *S. zooepidemicus*, also represent potential safety concerns (Liu et al. 2011). Obtaining highly purified HA is a challenging task, and given its increasing market demand, there is an urgent need for the development of a more efficient purification process.

Aqueous two-phase systems (ATPS) constitute a liquid-liquid extraction method formed by mixing two components beyond a critical concentration that results in two immiscible aqueous phases (González-Valdez et al. 2018; Sánchez-Trasviña et al. 2015). The most common ATPS types are those formed by polymer–polymer or polymer–salt mixtures. ATPS possess several advantages compared to conventional liquid–liquid extraction methods, such as a high-water content, low cost of components, ease of scaling up, process integration capacity, and high yields of the target product (Glyk et al. 2015; Gómez and Macedo 2019; Loureiro et al. 2017). When a solute is added into the ATPS, the partition between the two phases responds to several variables, namely the type and concentration of phase-forming components, molecular weight of polymers, system pH, temperature, and intrinsic physicochemical properties of the solute (Asenjo and Andrews 2011; Gu and Glatz 2007; Iqbal et al. 2016). The development of an optimal ATPS extraction stage is complex because of the number of interactions between the aforementioned factors governing the partition behaviour.

ATPS, given their aqueous environment and mild conditions, have been largely employed for the recovery and separation of a broad range of biomacromolecules, including proteins, enzymes, antibodies, peptides, and genetic material (Asenjo and Andrews 2011; Azevedo et al. 2009; Sánchez-Trasviña et al. 2019), as well as low molecular weight compounds with biological activity (Enriquez-Ochoa et al. 2020; Ghaffari et al. 2019; Simental-Martínez et al. 2014). Nevertheless, the application of ATPS for the separation of polysaccharides has been limited, mainly oriented as an extraction technique for polysaccharides from plant sources using ethanol-salt ATPS (Chen et al. 2016; Cheng et al. 2017; Wu et al. 2017; Zhang et al. 2018; Zhu et al. 2020), whereas the implementation of ATPS in the downstream processing of polysaccharides from microbial fermentations is practically unexplored. To date, there is only one study in the literature regarding the use of ATPS for the recovery of HA. Rajendran et al. (2016) implemented ATPS as a first step of the downstream processing of HA (> 1.8 MDa) produced in recombinant *L. lactis*. They explored polyethylene glycol (PEG) 6000-phosphate ATPS at different compositions and obtained a 97% recovery and 29.4% purity with an ATPS formed by 18% (w/w) PEG and 7% (w/w) potassium phosphate. Although protein impurities were removed to some extent, additional purification steps were necessary to achieve the required purity.

This study explored for the first time the use of PEG–citrate ATPS for the primary recovery and partial purification of HA produced in *S. zooepidemicus*. Sodium citrate was selected based on its biodegradable and non-toxic characteristics (Lu et al. 2010), which make citrate salts disposal on an industrial scale, as well as more eco-friendly compared to phosphate salts (Zafarani-Moattar et al. 2004). As a first step, the effects of PEG MW, tie-line length (TLL), and volume ratio (V_R_) on the partition behaviour and recovery of pure HA samples were investigated. Later, the PEG–citrate ATPS with the best recovery was selected and tested directly from *S. zooepidemicus* fermentation broth, where TLL, V_R_, and sample load were analysed.

## Materials and methods

### Materials

PEG of nominal molecular mass of 6000 (PEG6000, Cat. No. 81260) and 8000 g mol^−1^ (PEG8000, Cat. No. 89510), bovine serum albumin (BSA), sodium dodecyl sulphate (SDS), cetyltrimethylammonium bromide (CTAB), sodium chloride, and acetic acid were purchased from Sigma-Aldrich (St. Louis, MO, USA). A Pierce™ BCA Protein Assay Kit was obtained from ThermoFisher Scientific (Waltham, MA, USA). Sodium acetate anhydride was purchased from Fluka (Charlotte, NC, USA), and sodium citrate dihydrate from D.E.Q. (Monterrey, NL, Mexico). Cosmetic grade HA (0.8 MDa), from Chemico Especialidades Químicas (Guadalajara, Jal, Mexico), was used as a standard for HA quantification. All other reagents were of analytical grade. All substances were used without further purification. Milli Q-grade water was used to prepare all solutions.

Two pure *S. zooepidemicus*-derived HA samples of different molecular weights: 1–2 MDa (HA1.5) and 3–4 MDa (3.5HA), and a sample of *S. zooepidemicus* fermentation broth (MW ~ 3 MDa) were provided by BIOMENTUM SAPI de CV (Guadalajara, Jal, Mexico). Pure samples were received as solutions (0.15 M NaCl) and were used without further purification. All samples were stored at 4 °C prior to use.

### Preparation of crude extract

A sample of fermentation broth containing *S. zooepidemicus* cells was used to study the feasibility of using ATPS as a primary recovery and partial purification step of microbial HA. Upon arrival, the fermentation broth was diluted with a 20% (w/v) SDS solution at a 10:1 ratio (final SDS concentration of 2% w/v), and the mixture was stirred for 15 min to complete cell lysis and release the HA. Afterwards, the mixture was centrifuged at 10,000 rpm at 4 °C for 60 min using a Hermle Z 446 centrifuge (Labnet International, Edison, NJ, USA) to remove cell debris. The clarified supernatant was recovered and the sample, referred to as the crude extract, was stored at 4 °C prior to use.

### HA quantification

The concentration of HA was determined by the turbidimetric method using CTAB according to Song et al. (2009) with slight modifications. Briefly, 50 µL of standard or sample were mixed with 50 µL of 0.2 M acetate buffer (pH 6.0). The reaction was incubated at 37 °C for 5 min, followed by the addition of 100 µL of 2.5% (w/v) CTAB in 0.5 M NaOH (at 37 °C). The mixture was incubated at 37 °C for 10 min, and absorbance was measured instantly at 400 nm using a Synergy HT microplate reader (Biotek, Winooski, VT, USA). The calibration curve was prepared using HA standard solutions, ranging from 0 to 150 µg mL^−1^.

### Protein quantification

The amount of protein was quantified using the Pierce™ BCA Protein Assay Kit according to its microplate procedure. Briefly, 25 µL of standard or sample were mixed with 200 µL freshly prepared BCA working reagent (Reagent A-Reagent B, 50:1 ratio) and mixed gently for 30 s. The reaction was incubated at 37 °C for 30 min, then cooled at room temperature for 5 min, and absorbance was measured instantly at 562 nm using a microplate reader. The calibration curve was prepared using BSA solutions, ranging from 0.0 to 2.0 mg mL^−1^.

### Aqueous two-phase systems partition of pure HA

Pure HA samples were used to study the partition behaviour of HA in PEG–citrate ATPS. First, the effect of PEG MW (6000 and 8000 g mol^−1^) and TLL (23–43% w/w) at a V_R_ of 1.0 was analysed. Afterwards, the PEG MW with the best HA recovery was selected to evaluate different V_R_ (0.38 and 3.50) at the same TLLs. A total of 12 ATPS were tested.

ATPS were prepared by mixing determined weights of stock solutions of either PEG6000 (50% w/w) or PEG8000 (50% w/w) and sodium citrate (25% w/w) into clear, graduated 2-mL microcentrifuge tubes. The TLL and compositions of the ATPS were calculated based on the PEG–citrate binodal curves previously reported (Ghaffari et al. 2019) and summarised in Table 1. The sample was added to account for the 10% (w/w) of the ATPS, and the final mass of the ATPS was adjusted to 2.0 g with water. After all components were added, the tubes were thoroughly mixed for 15 min at room temperature. Phase separation was assisted by centrifugation at 10,000 rpm for 10 min at 25 °C using a 5417R centrifuge (Eppendorf, Hamburg, Germany). The volume of each phase was determined using tube graduation to calculate the ATPS V_R_ (top phase volume/bottom phase volume), and phases were carefully separated to quantify the HA concentration. ATPS with pure water instead of HA were used as blanks. Aliquots from both phases of the ATPS were diluted tenfold before carrying out the HA quantification protocol, since high salt concentrations prevent the formation of the CTAB-HA complex, thus impeding the analysis (Oueslati et al. 2014). The recovery percentage in each phase was calculated according to the following equation:

1 $$Recovery\left(\%\right)= \frac{{C}_{i}{V}_{i}}{{m}_{initial}}\times100,$$

where C_i_ is the concentration of HA in phase i (top or bottom), V_i_ is the volume of phase i, and m_initial_ is the initial mass of HA loaded into the system.

**Table 1:** Composition of polyethylene glycol (PEG)–citrate aqueous two-phase systems (ATPS) used in this work for the primary recovery of microbial hyaluronic acid (HA)

PEG MW (g mol^−1^)	V_R_	TLL (% w/w)	PEG (% w/w)	Citrate (% w/w)
6000	1.00	23	12.13	9.91
	1.00	35	16.45	10.48
	1.00	42	19.56	10.87
8000	0.38	24	6.95	11.80
	0.38	36	8.20	14.36
	0.38	43	7.74	16.36
	1.00	24	10.79	9.75
	1.00	36	16.31	10.94
	1.00	43	19.41	11.51
	3.50	24	17.62	7.20
	3.50	36	24.50	6.90
	3.50	43	28.91	6.85

MW: molecular weight; V_R_: volume ratio; TLL: tie-line length

### HA recovery and partial purification from crude extract

The feasibility of using PEG–citrate ATPS for the primary recovery and partial purification of HA was investigated using a crude extract. For this purpose, PEG8000–citrate systems (based on results with pure HA) were used. The effect of TLL (24, 36, and 43% w/w) and V_R_ (0.38 and 3.50) on HA recovery and purity were evaluated. Afterwards, the effect of increasing sample load (10, 12, and 14% w/w) on recovery and purity was further tested. ATPS were prepared following the same strategy as described in the previous section. In addition to HA quantification, protein concentration was also monitored, as the main contaminant present in the crude extract. Aliquots from both phases of the ATPS were diluted tenfold before carrying out the protein quantification protocol. Protein recovery was determined analogously with Eq. (1), and the HA purity percentage was calculated according to the following equation:

2 $$HA\;purity\left(\%\right)=\frac{{C}_{HA}}{{C}_{HA}+{C}_{P}}\times100,$$

where C_HA_ and C_P_ are the concentrations of HA and protein, respectively, in the bottom phase.

### Statistical analyses

Experiments were run as independent triplicates, and results were expressed as the mean ± standard error (SEM). Statistical analysis was performed using Minitab® (19.2020.1). One-way ANOVA and Tukey’s HSD test were implemented with a level of significance of 0.05 to assess significant differences between groups.

## Results

### Effect of TLL and PEG MW on HA recovery of pure samples

**Fig. 1:**
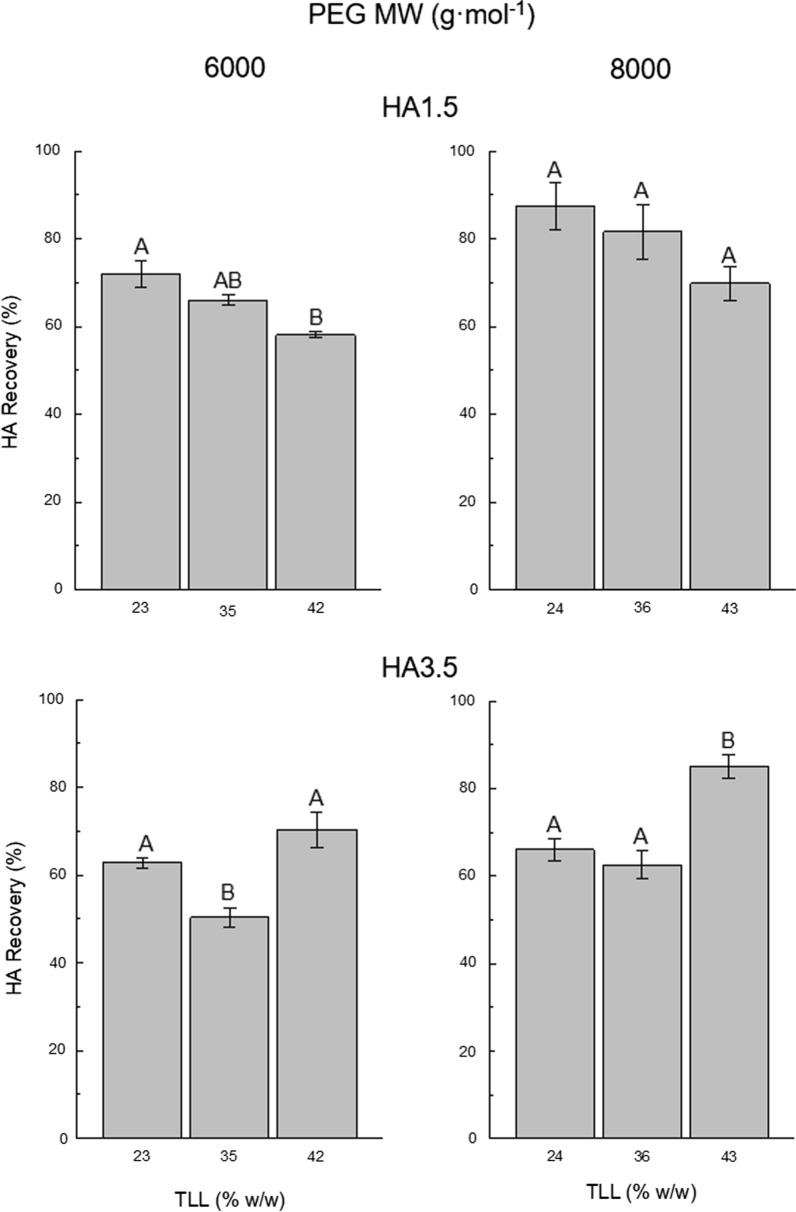
Effect of polyethylene glycol (PEG) molecular weight (MW, 6000 and 8000 g mol^−1^) and tie-line length (TLL, 23–43% w/w) on hyaluronic acid (HA) recovery in the bottom phase of PEG–citrate aqueous two-phase systems (ATPS). Two pure HA solutions of different molecular weights: 1–2 MDa (HA1.5) and 3–4 MDa (HA3.5) were evaluated. HA solutions were loaded to the ATPS at a final concentration of 10% (w/w). ATPS volume ratio (V_R_) was 1.0. Bars represent the sample mean ± SEM of experimental triplicates. Bars within the same graph that do not share the same uppercase letter are significantly different (p < 0.05)

HA partition behaviour in PEG–citrate ATPS had not been previously explored; therefore, its partition behaviour using PEG6000 and PEG8000 at three different TLLs and V_R_ 1.0 was first studied (Fig. 1). For this purpose, two pure HA sample solutions (Table 2) of different molecular weights were used: HA1.5 (MW: 1–2 MDa) and HA3.5 (MW: 3–4 MDa). HA migrated towards the salt-rich bottom phase regardless of the ATPS composition. Since negligible amounts of HA were detected in the PEG-rich top phase (data not shown), further analyses were focused on HA recovery in the bottom phase. Nevertheless, HA was continuously monitored in both phases.

**Table 2:** Composition of pure and crude extract of *Streptococcus equi* subsp. *zooepidemicus*-derived hyaluronic acid (HA) samples

Sample	HA MW (MDa)	HA concentration (g L^−1^)	Protein concentration (g L^−1^)
HA1.5	1–2	3.67	–
HA3.5	3–4	3.39	–
Crude extract	3.0	3.50	4.57

Samples were provided by BIOMENTUM SAPI de CV (Guadalajara, Mexico)

*MW* molecular weight

TLL had opposite effects on HA recovery between both samples (Fig. 1). For HA1.5, HA recovery was negatively affected by an increase in TLL, showing a significant change from 72.0 ± 3.1 to 58.1 ± 0.7% when going from TLL 23 to 42% (w/w) using PEG6000. Meanwhile for HA3.5, a significant increase of 20% on HA recovery was observed when going from TLL 35 to 42% (w/w) with PEG6000. This indicated that larger TLLs favoured the recovery of high molecular weight HA; however, it is difficult to state this conclusion since HA recovery was not significantly different between TLLs 23 and 42% (w/w). The maximum HA recovery for both samples was similar, around 70%; however, for HA1.5, it was found at the smallest TLL (23% w/w), while for HA3.5, it was found at the largest TLL (42% w/w). Since not all HA could be recovered in the bottom phase and its presence in the top phase was negligible, it is plausible that the remaining HA was retained at the interface; however, this region could not be analysed due to the impracticality of recovering HA from it.

An overall increase on HA recovery was noticed as PEG MW changed from 6000 to 8000 g mol^−1^ in both samples when comparing each pair of similar TLLs (23 vs. 24, 35 vs. 36, and 42 vs. 43% w/w), with an average increment on HA recovery of 12.1%. HA1.5 showed the same relationship between HA recovery and TLL for PEG8000 to that observed with PEG6000, whereas for HA3.5, the positive effect of TLL on HA recovery was more evident when using PEG8000; the highest HA recovery was attained at TLL 43% (w/w). In general, higher HA recovery percentages for both samples were obtained using PEG8000, although at different TLLs for each case. The highest HA recoveries reached were 87.4 ± 5.3 and 85.0 ± 2.7% for HA1.5 and HA3.5, respectively. Therefore, PEG8000 was established for the evaluation of the V_R_ effect.

### Effect of V_R_ on HA recovery of pure samples

**Fig. 2:**
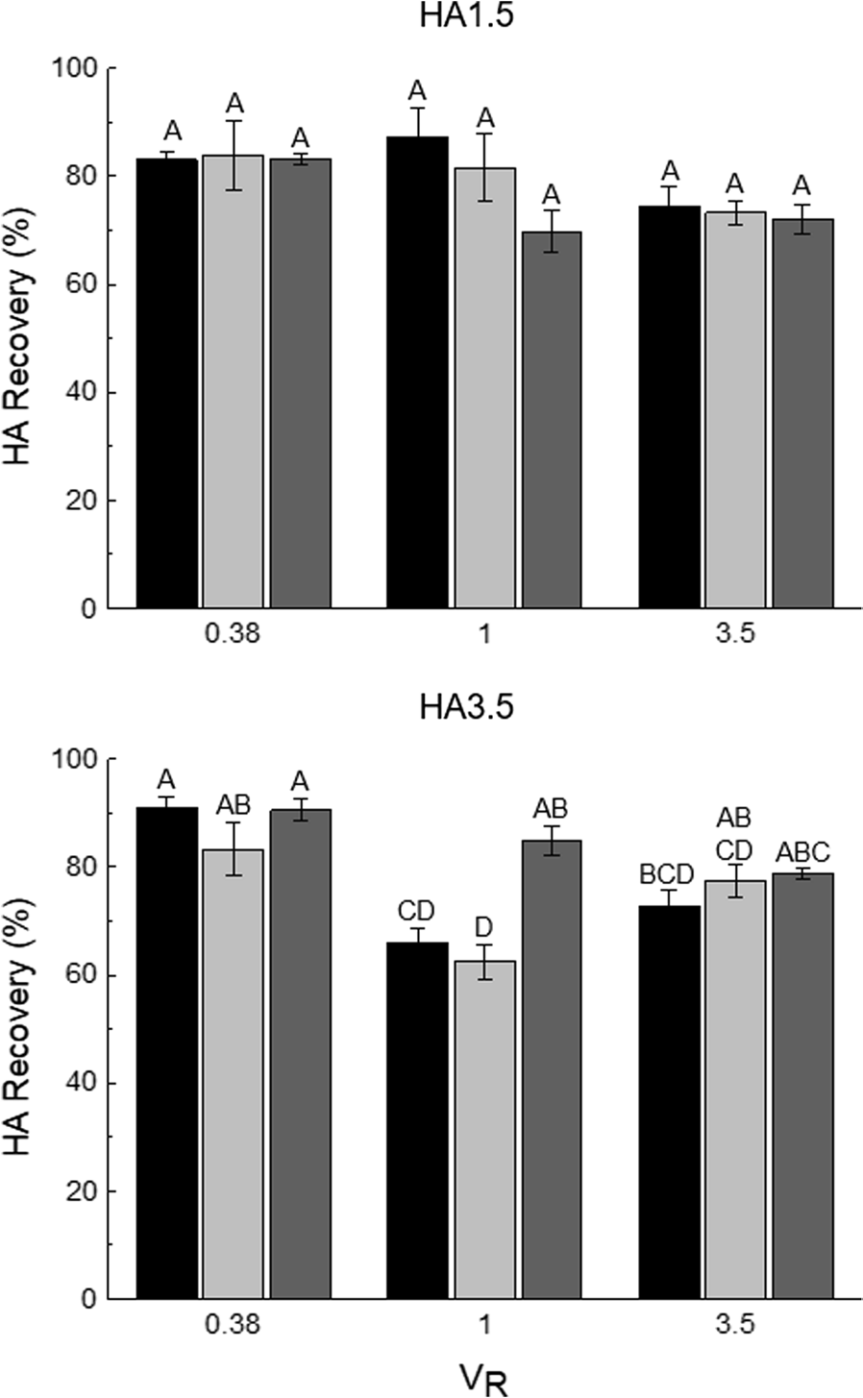
Effect of volume ratio (V_R_, 0.38, 1.00, and 3.50) and tie-line length (TLL, 24 (), 36 (), and 43 () % w/w) on hyaluronic acid (HA) recovery in the bottom phase of polyethylene glycol (PEG) 8000–citrate aqueous two-phase systems (ATPS). Two pure HA solutions of different molecular weights: 1–2 MDa (HA1.5) and 3–4 MDa (HA3.5) were evaluated. HA solutions were loaded to the ATPS at a final concentration of 10% (w/w). Bars represent the sample mean ± SEM of experimental triplicates. Bars within the same graph that do not share the same uppercase letter are significantly different (p < 0.05)

Following the selection of the PEG MW (8000 g mol^−1^), the effect of the ATPS V_R_ on HA recovery was evaluated by exploring two additional V_R_ (0.38 and 3.50) at the same established TLLs (24, 36, and 43% w/w). While a V_R_ of 1 indicated that the top and bottom phases were equal in volume, an ATPS with V_R _< 1 had a larger bottom phase volume and V_R _> 1, a larger top phase volume. Results on HA recovery of pure samples are shown in Fig. 2. In general, the same effect of V_R_ was observed for both samples. For instance, sample HA1.5 showed slight increases in HA recovery as V_R_ decreased, going from an average of 73.3% at V_R_ 3.50 to an average of 83.5% at V_R_ 0.38. For sample HA3.5, the effect of V_R_ on HA recovery was more evident, showing the highest recoveries (> 90%) at V_R_ 0.38 at TLL 24 and 43% (w/w). In fact, at TLL 24% (w/w), V_R_ 0.38 showed significant increases of 25 and 18% on HA recovery compared to V_R_ 1.0 and 3.50, respectively. Based on these observations, higher HA recoveries were favoured by lower V_R_ values.

### HA recovery and partial purification from a crude extract

**Fig. 3:**
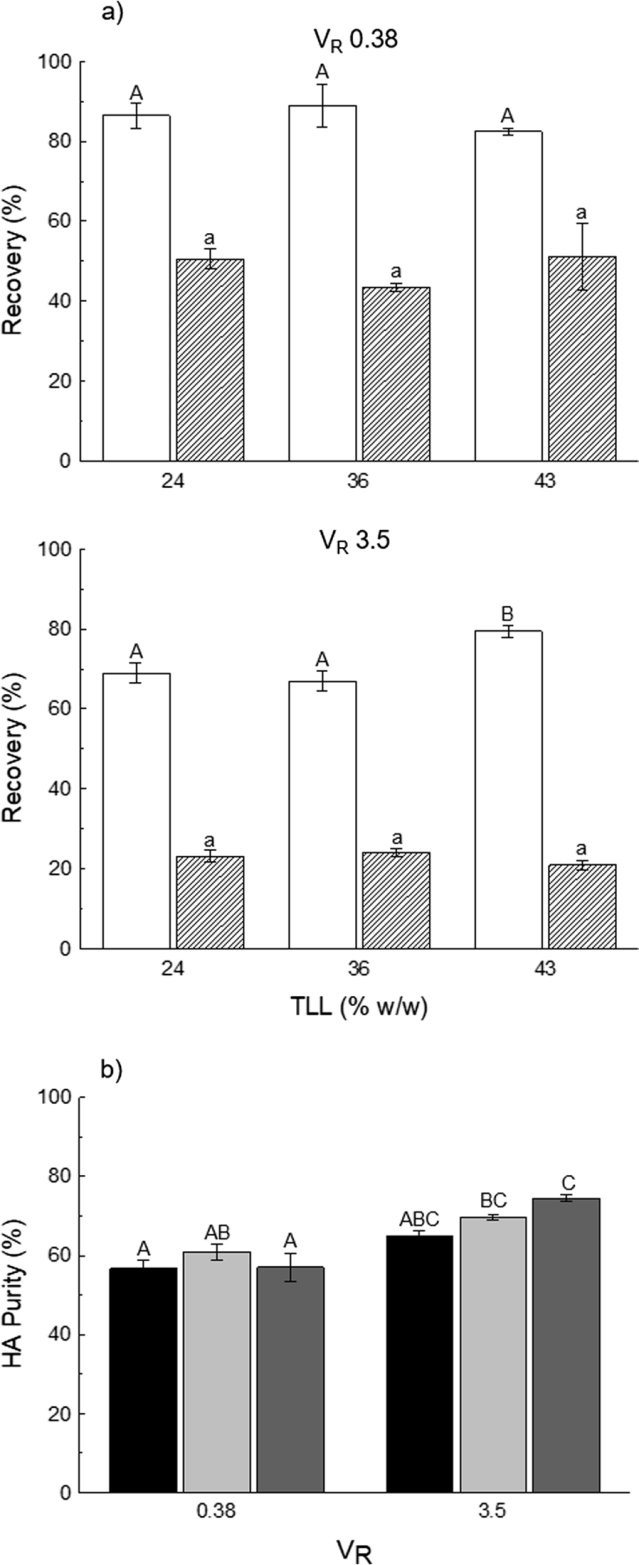
Effect of volume ratio (V_R_, 0.38 and 3.50) and tie-line length (TLL) on **a** hyaluronic acid (HA) (□) and protein () recovery in the bottom phase of polyethylene glycol (PEG) 8000–citrate aqueous two-phase systems (ATPS); and on **b** HA purity (TLL 24 (), 36 (), and 43 () % w/w). The crude extract (molecular weight: 3 MDa) was loaded to the ATPS at a final concentration of 10% (w/w). Bars represent the sample mean ± SEM of experimental triplicates. Bars within the same graph that do not share the same uppercase (HA) or lowercase (protein) letter are significantly different (p < 0.05)

A crude extract (MW ~ 3 MDa) was used to test the feasibility of implementing an ATPS as a primary recovery step and partial purification for microbial HA downstream processing. In addition to determining HA recovery, protein concentration in the bottom phase was also monitored in these ATPS to assess the capacity of protein removal and determine HA purity after separation. The initial characterisation of this sample (Table 2) revealed that HA purity after clarification was 43.4%. PEG8000–citrate ATPS were selected based on the greater HA recoveries seen with pure samples. Two V_R_ values (0.38 and 3.50) were evaluated at three TLLs (24, 36, and 43% w/w), and results are shown in Fig. 3. At V_R_ 0.38, HA and protein recovery were consistent across the three TLLs (no significant differences were found for both parameters), with average values of 85.9 and 48.4%, respectively, resulting in an average HA purity of 58.2%. HA recovery values were similar to those observed for pure sample HA3.5 (MW: 3–4 MDa) at V_R_ 0.38 (Fig. 2). Increasing the V_R_ to 3.50 resulted in overall HA loss. However, the consequent increase in the top phase volume favoured a drastic reduction of protein concentration in the bottom phase, near twofold compared to those of V_R_ 0.38, which translated into HA purities > 65% for all TLLs. From these observations, it was confirmed that HA recovery was higher at V_R_ 0.38, as was observed with pure samples, while protein removal in the bottom phase was higher at V_R_ 3.50. Based on HA purity results, the ATPS with the best performance was PEG8000–citrate, TLL 43% (w/w), and V_R_ 3.50 (HA recovery 79.4 ± 1.5%, HA purity 74.5 ± 0.8%). This system was selected for further evaluation.

### Effect of crude extract load on HA recovery and partial purification

**Fig. 4:**
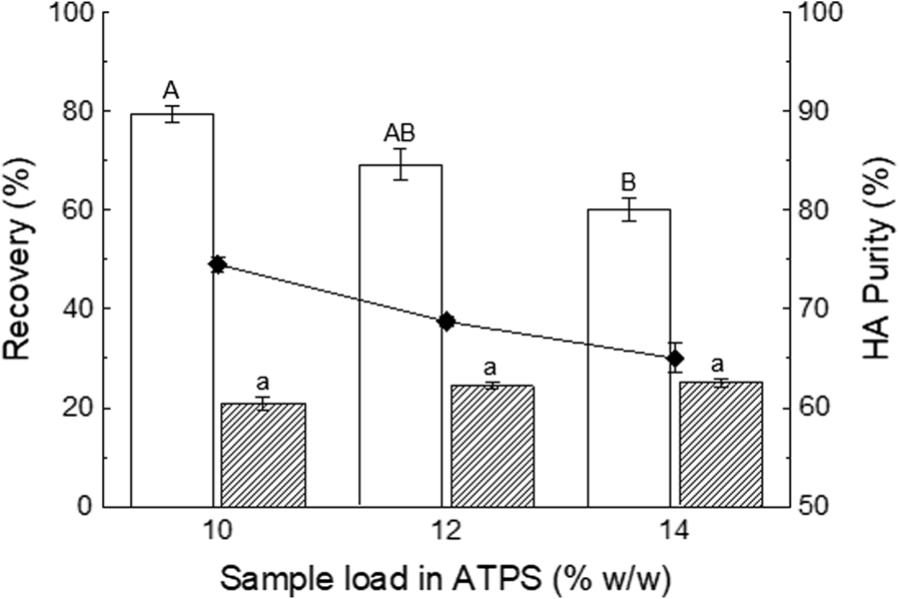
Effect of sample load (10, 12, and 14% w/w) of crude extract (molecular weight: 3 MDa) on hyaluronic acid (HA) (□) and protein () recovery in the bottom phase of polyethylene glycol (PEG)–citrate aqueous two-phase systems (ATPS); and on HA purity (◆). PEG molecular weight was 8000 g mol^−1^, TLL 43% (w/w) and V_R_ 3.50. Bars represent the sample mean ± SEM of experimental triplicates. Bars that do not share the same uppercase (HA) or lowercase (protein) letter are significantly different (p < 0.05)

Lastly, the effect of load capacity of the selected ATPS (PEG8000–citrate, TLL 43% w/w, V_R_ 3.50) was tested. Preliminary studies with pure samples showed that increasing sample load up to 14% (w/w) caused slight decreases in HA recovery (< 5%; data not shown); therefore, ATPS with a crude extract load up to 14% (w/w) were explored to assess process robustness. Figure 4 shows that protein recovery remained consistent (23.4% in average) at different crude extract loads (10, 12, and 14% w/w). However, HA recovery was drastically affected, as it decreased almost 20% when going from 10 to 14% (w/w) crude extract, which consequently affected HA purity. Although the sample load increase was minimal, it had a considerable negative impact on HA recovery and purity. Thus, a crude extract load of 10% (w/w) was established as the best.

## Discussion

HA has become an extraordinary biomaterial with multiple applications, especially in the fields of medicine and cosmetics. The formulation of HA-based products for these purposes requires a starting HA material with a high degree of purity, in compliance with clinical standards, to prevent undesired side-effects. One of the most common operations in the early stages of microbial HA downstream processing is precipitation by alcohols, usually ethanol or isopropanol in proportions from 1:1 to 3:1 v/v alcohol:broth and/or several consecutive steps. Although the toxicity of these short-chain alcohols is lower than other organic solvents, the fact of employing large volumes still represents a risk associated with their manipulation, environmental concerns, and costly disposals at industrial scales. Given their aqueous environment, ATPS represent an environmentally friendlier alternative than liquid extractions using organic solvents; moreover, their implementation in the downstream processing of high-value biomolecules produced in microbial fermentations is well documented. Thus, the purpose of this work was to test the feasibility, based on its product recovery and impurity removal capacity, of PEG–citrate ATPS as a primary recovery step in the production of *S. zooepidemicus*-derived HA.

Regardless of the ATPS composition, HA of both MWs was partitioned predominantly to the salt-rich bottom phase, in agreement with previous findings (Rajendran et al. 2016). This partition behaviour could be explained by different phenomena. Since both polymers have high MWs (6 and 8 kDa for PEG, 1–2 and 3–4 MDa for HA), a steric hindrance effect occurs. The free volume in the PEG-rich phase is not large enough for HA molecules to migrate to the top phase; thus, HA is partitioned towards the bottom phase. A similar effect occurs in polymer-polymer (e.g., PEG-dextran) ATPS, in which steric exclusion drives the separation of the two polymers into different phases (Asenjo and Andrews 2011). Furthermore, the solubility of HA in either PEG-rich or salt-rich environments also influences its partitioning. The pKa of the carboxyl groups in HA is between 3 and 4 (Dosio et al. 2016); thus, at the pH of the salt-rich bottom phase (~ 8.0) (Ghaffari et al. 2019), the carboxyl groups are deprotonated, making HA a polyanion with preference for the more hydrophilic phase, where ionic species are easily solvated by free water molecules. In addition, the partitioning of biomolecules in ATPS is also driven by electrostatic interactions between the biomolecule and the components of the two phases (Yang et al. 2010). Particularly in PEG-salt ATPS, the salt-rich bottom phase bears a more negative charge than the PEG-rich top phase, as a consequence of an accumulation of anions; thus, proteins with a positive net charge tend to partition into the bottom phase, while negatively charged proteins are directed to the top phase (Azevedo et al. 2009; Cavalcanti et al. 2006; Herculano et al. 2012). Although different salts may affect electrostatic interactions in ATPS in different ways. In a PEG1000–ammonium sulphate ATPS, 500 kDa sulphated dextran partitioned predominantly to the PEG-rich top phase, possibly due to sulphate group repulsion. However, when this top phase was recovered and a different salt, such as sodium citrate, was added to induce the formation of a second ATPS, sulphated dextran partitioned predominantly to the salt-rich bottom phase (Du et al. 2018). Sulphated dextran and HA are both examples of high molecular weight negatively charged polysaccharides.

The partitioning of polysaccharides in PEG-salt ATPS is a complex process affected differentially by all system parameters. In PEG6000–citrate and PEG8000–citrate ATPS at V_R_ 1.0, TLL had opposite effects on HA recovery depending on the MW of the polysaccharide. In general, as TLL increased, the free volume in the top phase was reduced (Sánchez-Trasviña et al. 2019); thus, HA was forced to migrate towards the bottom phase as TLL increased. This was observed for sample HA3.5 but not for HA1.5. By studying the partitioning behaviour of dextran of different MWs in PEG-ammonium sulphate ATPS at different TLLs, Du et al. (2018) observed that neither TLL nor dextran MW influenced the recovery of dextran in the bottom phase in a specific way. Therefore, TLL and HA MW may not have a straightforward influence on HA recovery.

Conversely, the favourable effect of PEG MW on HA recovery may also be explained by the volume exclusion effect. As the PEG MW increased, the occupied volume by the polymer increased as well (Silva et al. 2018) and forced the migration of HA to the bottom phase, as previously mentioned. Moreover, increasing the PEG MW increased the hydrophobicity of the top phase (Iqbal et al. 2016), which in consequence promoted HA, being highly water-soluble, to migrate towards the bottom phase.

V_R_ had a more precise effect on HA recovery, with low V_R_ values enhancing recovery. A V_R _< 1 implied a bottom phase that was larger in volume than the top phase; this represented more available space to solubilise the same amount of HA added to the system, overcoming phase saturation issues (Gómez-Loredo et al. 2014). Moreover, only at V_R_ 1.0 was HA recovery sensitive to TLL, whereas at either V_R_ 0.38 or 3.50, recovery was not affected by TLL. This is practical in the way that a great HA recovery can be obtained with an ATPS with a minimum concentration of PEG and citrate if V_R_ was held at 0.38. ATPS at V_R_ 0.38 and 3.50 were more easily disturbed than ATPS at V_R_ 1.00, which was observed as a slight cloudiness in the top phase during manual separation. Therefore, these systems require special care during handling to prevent re-mixing of phases and consequent recovery loss. This phenomenon could be attributed to the fact that the compositions of these systems are closer to the binodal curve of the PEG8000–citrate ATPS, which denotes the boundary between the one- and two-phase regions.

When working with the crude extract, V_R_ 0.38 displayed greater HA recoveries than V_R_ 3.50, as seen with pure samples. Overall, HA recovery was lower at both V_R_ when comparing the results to sample HA3.5, which was closer in MW to the HA in the crude extract. The crude extract is the viscous cell-free fermentation broth, with proteins as the major contaminant. In fact, these systems presented a visible interface with suspended particles, which was thicker as TLL increased. This thick interface helped to maintain phase separation during handling. Nevertheless, the presence of proteins above > 2 mg mL^−1^ hamper the recovery of HA by phase saturation (Benavides and Rito-Palomares 2008), explaining the differences between HA recoveries in pure samples and the crude extract. Protein removal from the bottom phase was considerably greater when the PEG/salt ratio increased (V_R_ 3.50), reducing by half the protein concentration compared to V_R_ 0.38. Proteins have a general affinity towards PEG molecules; therefore, by increasing the volume of the PEG-rich phase, a greater number of proteins were able to migrate to the top phase. The purity of HA recovered from the PEG8000–citrate, TLL 43% (w/w), V_R_ 3.50 ATPS was 31% higher than the HA in the crude extract. This increment in HA purity was greater than that obtained in a previous report where PEG6000-phosphate systems were used (Rajendran et al. 2016). However, in the work of Rajendran et al. (2016) crude extract load was considerably larger (between 64.0 and 80.5% w/w, depending upon the PEG/salt composition), and nucleic acids were also quantified as impurities.

Finally, the robustness of the selected ATPS (PEG8000–citrate TLL 43% w/w, V_R_ 3.50) was tested by increasing the amount of crude extract loaded into the system. However, this translated into a considerable HA loss even at 14% (w/w) crude extract. Given the low volume of the bottom phase of this system, phase saturation could be reached more easily. Moreover, not only the amount of HA but also contaminants increased at higher crude extract loads, which may have hindered the recovery of HA, as already discussed. In fact, protein recovery in the bottom phase remained constant from 10 to 14% (w/w) crude extract; this suggested that there are some proteins in the mixture that present a higher affinity towards the salt-rich bottom phase than HA, thus competing with the polysaccharide for the same available space.

Despite the advantages that ATPS offer as an extraction technique, their implementation in the downstream processing of high-value microbial polysaccharides is poorly explored. This work investigated for the first time the potential of PEG–citrate ATPS for the primary recovery and partial purification of HA produced in *S. zooepidemicus*. Screening different system parameters (PEG MW, TLL, V_R_, and sample load) demonstrated that PEG MW and system V_R_ were key factors that influenced the recovery of HA, which was enhanced with PEG of high MW (8000 g mol^−1^) and V_R_ 0.38. Increasing the amount of crude extract loaded into the system critically diminished HA recovery and purity, probably because of the large concentration of proteins. Using PEG8000–citrate, TLL 43% (w/w), and V_R_ 3.50 ATPS, HA was recovered at 79.4% from the cell-free fermentation broth, with a purity of 74.5%. Moreover, the low salt concentration of this system (6.85% w/w) was advantageous in the sense that fewer diafiltration steps would be required to desalt, if needed, HA in further downstream. It would be interesting to evaluate if PEG of a higher MW could further improve HA recovery from what was obtained in this study; however, PEG viscosity increases with MW, as does its cost, therefore, technical, and economical feasibilities must be considered for large-scale purification. Further studies should also be oriented to the integration of the ATPS step in the downstream processing of HA to evaluate overall yield and purity. In the context of the purification of microbial HA, PEG–citrate ATPS represent an attractive, easy to scale up alternative as a primary step to reduce the number of, or even replace, alcohol precipitation steps. Additionally, by using sodium citrate as the phase-forming salt, environmental concerns regarding its disposal were addressed.

## Data Availability

The authors can confirm that all relevant data are included in the article.
